# Assessing the impact of the COVID-19 pandemic on trends of select travel-acquired enteric illnesses in Canada

**DOI:** 10.14745/ccdr.v51i05a02

**Published:** 2025-05-01

**Authors:** Lauren Rusk, Russell Forrest, Meghan Hamel

**Affiliations:** 1 Centre for Food-Borne, Environmental and Zoonotic Infectious Diseases, Public Health Agency of Canada, Guelph, ON

**Keywords:** COVID-19, enteric illness, travel-acquired illness, travel, trends

## Abstract

**Background:**

Millions of Canadians contract enteric illnesses each year, many of which are acquired during, or are otherwise associated with, international travel. As the number of Canadians travelling fluctuates throughout the year, a corresponding change in the number of travel-acquired enteric illnesses was expected. A change in the number of travel-acquired enteric illnesses was also expected during the COVID-19 pandemic restrictions.

**Objective:**

This study aims to explore trends in the number and distribution of select travel-acquired enteric infections in Canada, from May 2017 to April 2023.

**Methods:**

To evaluate trends, Student’s t-tests and negative binomial regression modelling were conducted. Percent changes and relative risks were calculated to assess the impact of the pandemic on travel-acquired enteric illnesses.

**Results:**

Findings demonstrated a seasonal peak in the number of reported travel-acquired enteric illnesses during the winter and spring pre- and post-pandemic travel restrictions (May 2017–February 2020 and September 2021–April 2023). Additionally, there was a decrease in the number of travel-acquired enteric illnesses added to enteric illness travel clusters with cases in more than one province or territory (multi-jurisdictional) during and after the lifting of COVID-19 travel restrictions. However, cases reported post-travel restrictions had a higher risk of being added to a multi-jurisdictional enteric illness travel cluster compared to the pre-travel restriction phase.

**Conclusion:**

Nonessential travel restrictions and changes in the healthcare-seeking behaviours due to the pandemic likely account in part for the change in the number of travel-acquired enteric illnesses observed while travel restrictions were implemented and after they were lifted. Further research is required to explain the increased risk of illnesses being added to multi-jurisdictional enteric illness travel clusters after the lifting of travel restrictions compared to pre-COVID-19.

## Introduction

Each year, millions of Canadians experience enteric illnesses, of which approximately 25% are acquired during international travel ( ((1))). Research suggests that the incidence of travel-acquired enteric illness correlates with the number of travellers, peaking during periods of high travel activity ( ((2,3))). In Canada, this tends to occur during the winter months and previous studies in Ontario and British Columbia have confirmed this increase at the provincial level ( ((4–6))).

In March 2020, the World Health Organization declared the SARS-CoV-2 virus outbreak a pandemic. This led to countries implementing various public health measures to try and curb its spread ( ((7))). These measures also impacted the transmission of other infectious diseases. For example, multiple countries reported a decrease in the observed incidence of enteric infections ( ((8–11))). One explanation for this decrease may be a corresponding decline in the number of travel-acquired cases of enteric illness due to the implementation of nonessential travel restrictions ( ((8,9,11,12))). In Canada, nonessential travel restrictions were in effect from March 2020–August 2021 ( ((13,14))). During this period, a reduction in the number of reported travel-acquired enteric illness cases was observed ( ((8))). However, trends and a more detailed analysis of the changes in the risk of travel-acquired enteric infections before, during and after this period remain insufficiently studied.

After nonessential travel restrictions were lifted, the phrase “revenge travel” emerged to describe the expected surge in travel activity as people rescheduled cancelled plans ( ((15))). It was hypothesized that this might lead to an increase in travel-acquired enteric infections. Currently, little is known about the extent of the “revenge travel” phenomenon and its potential effects on the number of travel-acquired enteric illness cases in Canada.

This study aims to analyze trends in travel-acquired enteric infections in Canada at the national level and assess how travel-acquired enteric infections were influenced by the imposition and removal of nonessential travel restrictions during the COVID-19 pandemic.

## Methods

### Data sources

Since 2017/2018, whole-genome sequencing (WGS) has been routinely performed in Canada on various enteric pathogens, including *Salmonella*, *Listeria*, *Escherichia coli* and *Shigella*, at the National Microbiology Laboratory or by a PulseNet Canada-certified provincial laboratory. Whole-genome sequencing data is shared with PulseNet Canada and compared nationally using whole-genome multi-locus sequencing typing (wgMLST) within a central BioNumerics v7.6.3 database (Applied Maths, United States). PulseNet Canada assigns cluster codes to *Salmonella*, *Listeria*, *E. coli* and *Shigella* clusters when two or more isolates (where at least one is clinical) group together within 10 wgMLST allele differences within a specified period. The criteria for common *Salmonella* serotypes (including *S*. Enteritidis, *S*. Heidleberg and *S*. Typhimurium) is three or more isolates grouping together within 10 wgMLST allele differences with at least two isolates within five alleles within a specified period. Clusters can be either single jurisdictional, with cases occurring in only one province/territory, or multi-jurisdictional, if cases occur in multiple provinces/territories. Any case that does not group within 10 wgMLST allele differences of another case is considered sporadic. As single-jurisdictional clusters and sporadic cases are not routinely investigated at the national level, these were excluded from our analyses.

Epidemiologists at the Public Health Agency of Canada (PHAC) review all multi-jurisdictional clusters and classify them to aid with follow-up. Only clusters classified as travel-related were included in our analyses. A cluster was deemed travel-related if 1) there was strong epidemiological evidence to suggest the illnesses were acquired while outside of Canada (i.e., people acquired an enteric illness while outside Canada but were tested in Canada upon their return), 2) the cluster was genetically related (i.e., within 10 wgMLST allele differences) to another previously classified travel cluster or 3) the serotype is not endemic to Canada (i.e., *S. typhi* and *S. enterica* serovar Paratyphi A) ( ((16))). At the time of our study, no multi-jurisdictional travel-related clusters of *Listeria* had been identified in Canada at the federal level. Therefore, only *Salmonella*, *E. coli* and *Shigella* were considered.

Using these criteria, we analyzed cases added to multi-jurisdictional *Salmonella* travel clusters in Canada from May 2017 to April 2023 and *E. coli* and *Shigella* travel clusters from June 2018 to April 2023 as a representation for all travelled-acquired cases of enteric illness. The date used for cases was the earliest of the following dates: 1) the isolation date, 2) the date the isolate was received for WGS or 3) the date the case was reported to PHAC.

Data for the number of Canadians travelling internationally was retrieved from a publicly available Statistics Canada dataset ( ((17))). These data were collected by the Frontier Counts program, which counts the number of individuals entering Canada. For this study, the number of Canadian residents returning from countries other than the United States was used. Travel to the United States was excluded due to the similarities in the Canadian and American food supply and because, to date, there has not been a multi-jurisdictional enteric illness cluster solely associated with travel to the United States identified in Canada.

### Statistical analyses

Three time periods were analyzed: pre-COVID-19 pandemic travel restrictions (before travel restrictions related to the COVID-19 pandemic were issued; May 2017–February 2020), COVID-19 pandemic travel restrictions (March 2020–August 2021) and post-COVID-19 pandemic travel restrictions (after the travel restrictions were revoked; September 2021–April 2023). Cases were assigned to these phases based on the earliest available date described above.

Trends in the number of cases of enteric illness added to multi-jurisdictional enteric illness travel clusters were assessed using two-sided Student’s t-tests to compare the mean monthly number of cases between phases. A negative binomial regression model was also employed. Negative binomial regression is suitable for modelling discrete count data that is left-censored at zero, which is common in epidemiological studies ( ( ( ((18))))). Variables included in model building were time (in one-month increments), meteorological season (winter=December–February, spring=March–May, summer=June–August, fall=September–November), the monthly number of Canadians travelling internationally and the presence of travel restrictions. Two-way interactions between independent variables were also assessed. Model selection was preformed using stepwise selection along with the Akaike Information Criterion (AIC) and likelihood-ratio test.

Percent changes in the number of travellers and cases between phases were evaluated. Relative risks (RR) were calculated to assess changes in the risk of cases being added to multi-jurisdictional enteric illness travel clusters, comparing the COVID-19 travel restrictions and post-travel restrictions phases to a pre-COVID-19 reference period of equal length.

All statistical analyses were conducted in Stata/MP 15 (Stata Corporation, United States) and utilized an alpha value of 0.05.

## Results

### Trends in travel-acquired enteric infections

**Figure 1:**
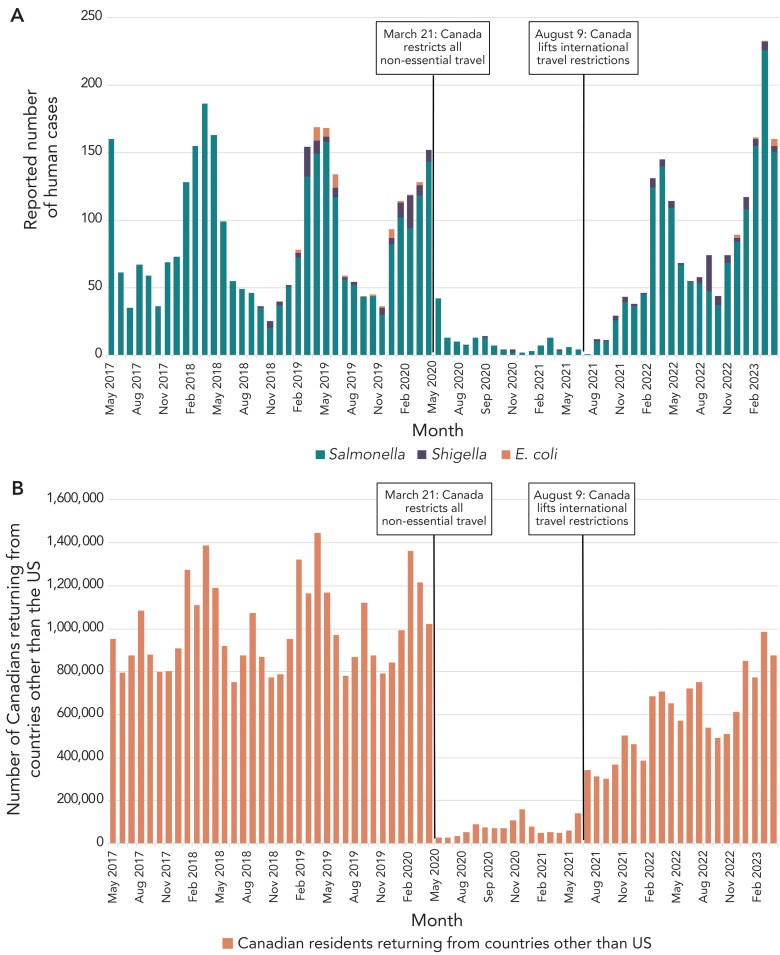
Trends in A) Canadian travel-acquired enteric infections added to multi-jurisdictional clusters and B) Canadians returning from international travel^a^, May 2017–April 2023 Abbreviations: *E. coli*, *Escherichia coli*; US, United States ^a^ Data were collected by the Frontier Counts program, which counts the number of individuals entering Canada. Data from countries other than the United States were used

From May 2017 to February 2020, the number of travel-acquired *Salmonella* infections added to multi-jurisdictional enteric illness travel clusters in Canada was significantly higher in February and March (*p*=0.0436 and *p*=0.0312, respectively; **Figure 1**) compared to other months. Travel-acquired cases of *Shigella* and *E. coli* did not exhibit any significant seasonal trends between June 2018 and February 2020. No seasonal trends were observed from March 2020 to August 2021 for all three pathogens while nonessential travel restrictions were in effect. From September 2021 to April 2023, the total number of monthly travel-acquired enteric infections added to multi-jurisdictional enteric illness travel clusters in Canada was significantly higher in March and April (*p*=0.0053 and *p*=0.0017, respectively). These increases correspond with increased international travel by Canadians during the winter and spring (Figure 1).

The negative binomial regression model that best fit the data (AIC=619.62) included all independent variables and the interaction term between time and nonessential travel restrictions (**Table 1**). The RR for a reported case of enteric illness being added to a multi-jurisdictional enteric illness travel cluster was higher during the winter (RR 1.32; 95% CI: 1.03–1.7) and spring (RR 2.26; 95% CI: 1.77–2.9) compared to the summer, with no significant difference in RR between the summer and fall.

**Table 1:** Model coefficients from the negative binomial regression model

Variable	β	Standard error	95% CI		Z value	*p-*value
			LL	UL		
Intercept	−192	57.87	−300.88	−86.64	−3.32	<0.001
**Meteorological season**						
Summer	Referent	-	-	-	-	-
Fall	−0.041	0.13	−0.29	0.2	−0.33	0.74
Spring	0.82	0.13	0.57	1.06	6.52	<0.001
Winter	0.28	0.13	0.031	0.53	2.25	0.025
Number of travellers (centred at 668,199.4)	1.36e-6	2.10e-7	9.31e-7	1.8e-6	6.49	<0.001
Time (measured in one-month intervals)	0.097	0.029	0.043	0.15	3.38	<0.001
**Restrictions on nonessential travel**						
No	Referent	-	-	-	-	-
Yes	3,273	529.7	2,261.5	4,327.81	6.18	<0.001
Interaction term between time and travel restrictions	−1.62	0.26	−2.14	−1.12	−6.18	<0.001

Abbreviations: CI, confidence interval; LL, lower limit; UL, upper limit; -, not applicable

### Impact of COVID-19 pandemic travel restrictions

**Figure 2:**
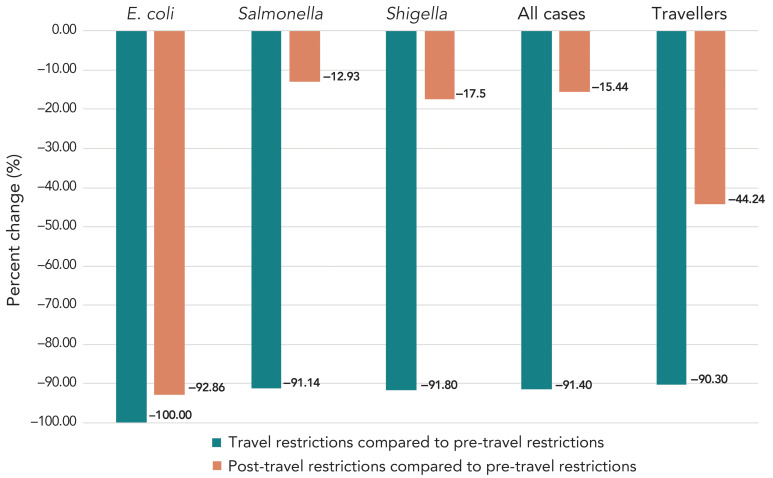
Percent change in the number of Canadians returning from international travel and the number of travel-acquired enteric infections added to multi-jurisdictional enteric illness clusters by pathogen between phases of COVID-19 travel restrictions Abbreviation: *E. coli*, *Escherichia coli*

Between June 2020 and August 2021, the number of Canadians returning from countries other than the United States decreased by 90.3% compared to the same period in 2018–2019 (1,447,595 travellers vs. 14,924,122 travellers). This decline in travellers corresponded with a 91.4% decrease in the number of reported cases of enteric illness added to multi-jurisdictional travel clusters (100 cases vs. 1,163 cases), which was observed across all three pathogens (**Figure 2**). Despite the overall decrease, the RR of reported cases of enteric illness being added to multi-jurisdictional travel clusters during the COVID-19 travel restriction phase did not change compared to the pre-COVID-19 travel restrictions phase (**Table 2**).

**Table 2:** Relative risks by pathogen, between phases of COVID-19 pandemic travel restrictions^a^

Comparison	Pathogen							
	*Salmonella*		*Shigella*		*Escherichia coli*		Overall	
	RR (95% CI)	*p*-value	RR (95% CI)	*p*-value	RR (95% CI)	*p*-value	RR (95% CI)	*p*-value
COVID-19 travel restrictions to pre-COVID-19 travel restrictions	0.91 (0.74–1.13)	0.44	0.85 (0.34–2.10)	1	0 (0.0–0.0)	0.11	0.89 (0.72–1.09)	0.26
Post-COVID-19 travel restrictions to pre-COVID-19 travel restrictions	1.57 (1.45–1.70)	<0.001	1.46 (1.11–1.92)	0.007	0.34 (0.16–0.73)	0.003	1.53 (1.42–1.64)	<0.001

Abbreviations: CI, confidence interval; RR, relative risk

^a^ Reference period: pre-COVID-19 travel restriction period

### Post-COVID-19 pandemic nonessential travel restrictions

Following the lifting of international travel restrictions in August 2021, both international travel by Canadians and the reported number of enteric infections added to multi-jurisdictional travel clusters increased, but overall neither had returned to pre-pandemic levels (Figure 1). From September 2021 to February 2023, international travel was 44.2% lower compared to the 2018–2020 reference period (10,206,099 travellers vs. 18,304,311 travellers). Similarly, the number of reported enteric infections added to multi-jurisdictional travel clusters decreased by 15.4% (1,309 cases vs. 1,548 cases). Despite this, the RR of enteric illnesses added to multi-jurisdictional travel clusters was higher during the post-COVID-19 travel restriction phase compared to the pre-COVID-19 travel restriction phase, primarily due to an increased risk of travel acquired salmonellosis (Table 2).

## Discussion

Anecdotal evidence suggested that travel-acquired enteric illnesses in Canada peak during the winter months; however, this had not yet been confirmed nationally. This study finds that indeed international travel by Canadians is highest from January to April, corresponding with a peak in the reported number of travel-acquired enteric illnesses added to multi-jurisdictional travel clusters. This seasonal pattern aligns with trends observed in other countries, where travel-acquired enteric infections peak with increased travel activity ( ((2,3))). In Canada, this peak is primarily driven by an increase in the reported number of *Salmonella* infections, consistent with provincial-level trends observed in Ontario ( ((4,5))).

The COVID-19 pandemic prompted the Canadian government to introduce public health measures aimed at reducing the spread of the SARS-CoV-2 virus. These measures helped to reduce the incidence of not only COVID-19 but many other infectious diseases, including enteric diseases ( ((8–11))). Several hypotheses have been proposed for the reduction in enteric illness incidence rates during the pandemic including changes in exposure-causing behaviours, changes in healthcare-seeking behaviours and a decrease in international travel. In March 2020, Canada imposed restrictions on nonessential international travel, which remained in effect until August 2021 ( ((13,14))). These restrictions reduced international travel by Canadians by 90.3% compared to the pre-pandemic reference period. As approximately 25% of enteric infections in Canada are travel-related ( ((1))), a reduction in enteric infections was anticipated and confirmed, with a 91.41% decrease in reported travel-acquired enteric infections. However, the absence of a significant change in RR suggests that this decrease was likely due to less international travel rather than changes in the risk of contracting an enteric illness abroad.

As the COVID-19 travel restrictions ended, many expected a spike in travel activity as individuals capitalized on the re-established ability to travel. We hypothesized that there would be an increase in international travel by Canadians and a corresponding increase in the number of travel-acquired enteric infections. Contrary to this, travel by Canadians was slow to rebound completely, with a 44.24% reduction in the number of Canadian travellers compared to pre-COVID-19 levels. Moreover, the number of reported travel-acquired enteric infections added to multi-jurisdictional travel clusters decreased by 14.76%. Despite this decline, the RR of 1.53 (95% CI: 1.42–1.64) suggests a higher risk of travel-acquired cases of enteric illness being added to multi-jurisdictional clusters during this period. It is possible that, due to the COVID-19 pandemic, Canadians are now more health-conscious and aware of the health risks associated with international travel. Furthermore, given that many symptoms of enteric infections mirror those of COVID-19, more ill Canadians may have sought a medical diagnosis upon returning to Canada during this period out of fear that they had contracted COVID-19. This would result in cases of enteric illness being reported that historically may have gone undetected. Another possible explanation is that there were changes in the travel destinations of Canadians after the pandemic. As different countries have differing rates of enteric disease, this also could have contributed to the increased risk observed.

### Limitations

There are some limitations to this study that must be taken into consideration. First, the reported number of travel-acquired enteric infections used underestimates the true burden of travel-associated enteric illness in Canada. Our sampling frame consisted of cases of enteric illness for which a specimen was submitted for testing and WGS, and subsequently assigned to a multi-jurisdictional cluster. Cases of enteric illness often go unreported as many individuals never submit a specimen for testing and are therefore not captured by public health surveillance systems. Additionally, during the pandemic, laboratory testing for COVID-19 was prioritized, which may have reduced testing for enteric pathogens and resulted in further underreporting during this period. Furthermore, cases that were not part of a multi-jurisdictional enteric illness cluster were excluded as they are not routinely investigated at the federal level in Canada.

Additionally, when providing potential explanations for the increased risk since the lifting of travel restrictions, we could not assess whether the travel destinations of Canadians had changed. Therefore, it is not possible to conclude whether changes in travel behaviours amongst Canadians may have contributed to the increased RR.

Finally, misclassification is a concern. At the national level, epidemiologists review exposure details for cases, including whether cases travelled outside of Canada during their exposure period. If a number of cases in a multi-jurisdictional enteric illness cluster report international travel to the same destination, the cluster is classified as travel-associated. Subsequent cases added to the cluster are assumed to be travel-acquired based on their genetic relatedness, but this is generally not confirmed via exposure data. Similarly, clusters identified within 10 wgMLST allele differences to a cluster that was previously classified as a travel cluster are classified as travel-acquired without confirmation via exposure data. Therefore, it is possible that some cases included in this study may not be travel-acquired even though they are in a cluster that is classified as travel-associated. Additionally, due to the limited nature of the exposure information available at a national level, the immigration status of cases is often unknown, which may result in some cases of enteric illness being misclassified. Finally, cases were aggregated by month and categorized into phases based on the earliest date available for each case. All the dates used occurred after the cases’ symptom onset date, which may have caused the case to be misclassified into the wrong month. Future studies should apply quantitative methods to evaluate the impact of potential misclassification and other systemic errors.

## Conclusion

Identifying peak periods for travel-acquired enteric illnesses in Canada allows for timely deployment of public health resources and targeted messaging to increase awareness and reduce travel-related risks. The findings of this study highlighted seasonal increases in the number of travel-acquired enteric infections added to multi-jurisdictional enteric illness travel clusters during the winter and spring months. The reduction in cases during the pandemic is likely due to fewer people travelling during this time. While this helps clarify the impact of COVID-19 on these infections, further research is needed to understand the heightened risk during the post-pandemic travel restriction phase.
